# Nominata 2025

**DOI:** 10.61622/rbgo/2025nominata02025

**Published:** 2026-02-20

We wish to thank everyone who contributed to the edition of the Revista Brasileira de Ginecologia e Obstetrícia - RBGO volume 47, year 2025, especially the authors and reviewers whose work and opinions were essential to maintain the scientific and methodological rigor of the published articles.

**Albaro Nieto-Calvache,** Fundacion Valle del Lili, Cali, Valle del Cauca, Colombia

**A. Seval Ozgu-Erdinc,** Reproductive Endocrinology Department, University of Health Sciences, Dr. Zekai Tahir Burak Health Practice Research Center,Ankara, Turkey

**Abiodun S Adeniran,** University of Ilorin, Ilorin, Kwara, Nigeria

**Adauto Dutra,** Universidade Federal Fluminense, Niterói, RJ, Brazil

**Adeylson Ribeiro,** Fundação Oncocentro de São Paulo, São Paulo, SP, Brazil

**Adhemar Longatoo-Filho,** Hospital do Câncer de Barretos, Barretos, SP, Brazil

**Adrian Lim,** Imperial College London, London, UK

**Adriana Campaner,** Irmandade da Santa Casa de Misericordia de São Paulo, São Paulo, SP, Brazil

**Adriana Carbonel,** Universidade Federal de São Paulo, São Paulo, SP, Brazil

**Adriana Gualda Garrido,** Nexus, Brasilia, DF, Brazil

**Adriana Luz,** Universidade Estadual de Campinas, Campinas, SP, Brazil

**Adriana Pedro,** Universidade Estadual de Campinas, Campinas, SP, Brazil

**Adriana Saur,** Centro Universitário Barão de Mauá, Ribeirão Preto, SP, Brazil

**Adriana Yoshida,** Universidade Estadual de Campinas, Campinas, SP, Brazil

**Adriani Galão,** Universidade Federal do Rio Grande do Sul, Porto Alegre, RS, Brazil

**Adsom Vale,** Universidade Federal do Rio Grande do Norte, Natal, RN, Brazil

**Ahmed Abbas,** Assiut University, Egypt

**Ahmed Maged,** Kasr Alainy Medical School, Cairo, Egypt

**Ahmed Reda,** Ain Shams University Faculty of Medicine, Square, Egypt

**Airton Stein,** Universidade Federal de Ciências da Saúde de Porto Alegre, Porto Alegre, RS, Brazil

**Akinyemi Akintayo,** Ekiti State University, Ado Ekiti, Nigeria

**Alan Hatanaka,** Universidade Federal de São Paulo, São Paulo, SP, Brazil

**Albaro Nieto-Calvache,** Hospital Universitario Fundación Valle del Lili, Valle del Cauca, Colombia

**Alberto Carlos Moreno Zaconeta,** Universidade de Brasília, Brasília, DF, Brazil

**Alberto Frisoli Jr.,** Universidade Federal de São Paulo, São Paulo, SP, Brazil

**Alberto Madeiro,** Universidade Estadual do Piaui, Floriano, PI, Brazil

**Alberto Palomino,** Hospital Clinico San Borja Arriaran, Santiago, Chile

**Alberto Trapani Jr.,** Universidade Federal de Santa Catarina, Florianópolis, SC, Brazil

**Alberto Zaconeta,** Universidade de Brasilia, Brasilia, DF, Brazil

**Alessandra Caputo,** Universidade de Uberaba, Uberaba, MG, Brazil

**Alessandra Cristina Marcolin,** Universidade de São Paulo, Ribeirão Preto, SP, Brazil

**Alessandro Cavalcanti,** Universidade Estadual da Paraiba, Campina Grande, PB, Brazil

**Alessandro Favilli,** University of Perugia, Perugia, Italy

**Alex Souza,** Instituto de Medicina Integral Professor Fernando Figueira, Recife, PE, Brazil

**Alexandre Delgado,** Universidade Federal de Pernambuco, Recife, PE, Brazil

**Alexandre Rocha,** Universidade Federal de Ciências da Saúde de Porto Alegre, RS, Brazil

**Alexandrina Maria Cardoso,** Escola Superior de Enfermagem do Porto, Porto, Portugal

**Ali Cetin,** Cumhuriyet University Faculty of Medicine, Sivas Province, Turkey

**Alice Zelmanowicz,** Universidade Federal do Rio Grande do Sul, Porto Alegre, RS, Brazil

**Alim Demian,** Universidade Federal de Minas Gerais, Belo Horizonte, MG, Brazil

**Aline Andrade,** Universidade de São Paulo, Ribeirão Preto, SP, Brazil

**Aline Brilhante,** Universidade de Fortaleza, Fortaleza, CE, Brazil

**Aline Santiago,** Universidade Estadual Paulista Julio de Mesquita Filho, Botucatu, SP, Brazil

**Álvaro Luiz Lage Alves,** Universidade Federal de Minas Gerais, Belo Horizonte, MG, Brazil

**Amanda Dantas,** Universidade Estadual de Campinas, Campinas, SP, Brazil

**Amanda Ferracini,** Universidade Estadual de Campinas, Campinas, SP, Brazil

**Amaxsell de Sousa,** Universidade Federal do Rio Grande do Norte, Natal, RN, Brazil

**Amilcar Barreta,** Universidade Estadual de Campinas, Campinas, SP, Brazil

**Amir Shamshirsaz,** Baylor College of Medicine, Houston, Texas, USA

**Ana Bianchi,** Hospital Pediatrico Pereira Rossell, Montevideo, Uruguay

**Ana Caetano,** Universidade de São Paulo, São Paulo, SP, Brazil

**Ana Carolina Camargo,** Faculdade de Medicina de Jundiai, Jundiaí, SP, Brazil

**Ana Carolina Rosa e Silva,** Universidade de São Paulo, Ribeirão Preto, SP, Brazil

**Ana Cristina Araújo,** Universidade Federal do Rio Grande do Norte, Natal, RN, Brazil

**Ana Cristina Freitas de Vilhena Abrão,** Universidade Federal de São Paulo, São Paulo, SP, Brazil

**Ana Cristina Pinheiro Fernandes Araujo,** Universidade Federal do Rio Grande do Norte, Natal, RN, Brazil

**Ana Gabriela Travassos,** Universidade do Estado da Bahia, Slvador, BA, Brazil

**Ana Katherine da Silveira Gonçalves de Oliveira,** Universidade Federal do Rio Grande do Norte, Natal, RN, Brazil

**Ana Livia Pascom,** Universidade Federal de São Paulo, São Paulo, SP, Brazil

**Ana Lucia Valadares,** Universidade Estadual de Campinas, Campinas, SP, Brazil

**Ana Luiza Lunardi Rocha,** Universidade Federal de Minas Gerais, Belo Horizonte, MG, Brazil

**Ana Luiza Rocha Lunardi,** Universidade Federal de Minas Gerais, Belo Horizonte, MG, Brazil

**Ana Maria Kondo Igai,** Universidade de São Paulo, São Paulo, SP, Brazil

**Ana Paula Costa,** Universidade Federal do Rio Grande do Norte, Natal, RN, Brazil

**Ana Paula Esteves-Pereira,** Fundacao Oswaldo Cruz, Rio de Janeiro, RJ, Brazil

**Ana Paula Kussler,** Universidade Federal do Rio Grande do Sul, Porto Alegre, RS, Brazil

**Ana Selma Picoloto,** Universidade Federal do Rio Grande do Sul, Porto Alegre, RS, Brazil

**Ana Tereza Pimenta,** Universidade de São Paulo, Ribeirão Preto, SP, Brazil

**Ana Zamarian,** Universidade Federal de São Paulo, São Paulo, SP, Brazil

**Anca Panaitescu,** Harris Birthright Research Centre for Fetal Medicine, King’s College Hospital, London, UK

**Anderson Silva,** Universidade de São Paulo, Ribeirão Preto, SP, Brazil

**André Cunha,** Universidade Federal de Goias, Goiânia, GO, Brazil

**André Hadyme Miyague,** Universidade Federal do Paraná, Curitiba, PR, Brazil

**Andre Luis Santos,** Universidade de Taubate, Taubate, SP, Brazil

**Andre Luiz Malavasi Longo de Oliveira,** Universidade de São Paulo, São Paulo, SP, Brazil

**André Miyague,** Universidade Federal do Paraná, Curitiba, PR, Brazil

**Andrea Betina Schmitt Palmieri,** Hospital Infantil Darcy Vargas, São Paulo, SP, Brazil

**Andréa Cronemberger Rufino,** Universidade Estadual do Piauí, Floriano, PI, Brazil

**Andréa da Rocha Tristão,** Universidade Estadual Paulista “Júlio de Mesquita Filho”, Botucatu, SP, Brazil

**Andrea Damin,** Universidade Federal do Rio Grande do Sul, Porto Alegre, RS, Brazil

**Andréa Nácul,** Hospital Fêmina, Porto Alegre, RS, Brazil

**Andrea Pires Souto Damin,** Universidade Federal do Rio Grande do Sul, Porto Alegre, RS, Brazil

**Andrea Rufino,** Universidade Estadual do Piaui, Floriano, PI, Brazil

**Andrea Silveira,** Universidade de São Paulo, Ribeirão Preto, SP, Brazil

**Andreisa Paiva,** Universidade Federal do Ceara, Fortaleza, CE, Brazil

**Angel Paternina-Caicedo,** Universidad de Cartagena, Cartagena, Bolívar, Colombia

**Angela Reichelt,** Hospital de Clínicas de Porto Alegre, Porto Alegre, RS, Brazil

**Angela Vinturache,** Oxford University Hospitals Foundation Trust, Oxford, Oxfordshire, UK

**Angélica Diniz,** Universidade Federal de Uberlandia, Uberlandia, MG, Brazil

**Angélica Nogueira Rodrigues,** Universidade Federal de Minas Gerais, Belo Horizonte, MG, Brazil

**Angélica Simone Silva,** Universidade de Pernambuco, Recife, PE, Brazil

**Anita Pimentel,** Universidade Federal do Rio Grande do Sul, Porto Alegre, RS, Brazil

**Anke Bergman,** Instituto Nacional de Cancer, Brasília, DF, Brazil

**Antonia Paula Marques de Faria,** Universidade Estadual de Campinas, Campinas, SP, Brazil

**Antônio Amorim Filho,** Universidade de São Paulo, São Paulo, SP, Brazil

**Antonio Carlos Zuliani de Oliveira,** Universidade Estadual de Campinas, Campinas, SP, Brazil

**Antonio Faria,** Università di Bologna, Bologna, Italy

**Antonio Nogueira,** Universidade de São Paulo, Ribeirão Preto, SP, Brazil

**Antonio Rodrigues Braga Neto,** Universidade Federal do Rio de Janeiro, Rio de Janeiro, RJ, Brazil

**Arda Isik,** Centro Médico da Universidade de Pittsburgh, Pitsburgo, Pensilvânia, USA

**Ariane Polidoro Dini,** Universidade Estadual de Campinas, Campinas, SP, Brazil

**Arlley da Silva,** Fetal Medicine Research Institute, London, UK

**Arn Migowski,** Instituto Nacional de Câncer, Rio de Janeiro, RJ, Brazil

**Arthur Antolini-Tavares,** Universidade Estadual de Campinas, Campinas, SP, Brazil

**Artur Dzik,** Hospital Pérola Byington, São Paulo, SP, Brazil

**Atakan Tanacan,** Hacettepe Universitesi Tip Fakultesi, Ankara, Turkey

**Atila Trape,** Universidade de São Paulo, São Paulo, SP, Brazil

**Atilio Di Spiezio Sardo,** Universidade de Nápoles Federico II, Napoli, Italy

**Augusto Benedeti,** Universidade de São Paulo, Ribeirão Preto, SP, Brazil

**Augusto Brandão,** Mater Dei Rede de Saúde, Belo Horizonte, MG, Brazil

**Barros Elvino,** Universidade Federal do Rio Grande do Sul, Porto Alegre, RS, Brazil

**Beatriz Paulina Ayala Quintanilla,** La Trobe University, Bundoora, Austrália

**Belmiro Pereira,** Universidade Estadual de Campinas, Campinas, SP, Brazil

**Beyza Ozcinar,** Istanbul School of Medicine, General Surgery Department, Istanbul University

**Brena Melo,** Faculdade Pernambucana de Saúde, Recife, PE, Brazil

**Brenno Belazi Nery de Souza Campos,** Universidade Estadual de Campinas, Campinas, SP, Brazil

**Bruno Borghese,** Université Paris Descartes Faculté de Médecine, Paris, França

**Bruno Ramalho,** Hospital Sírio-Libanês, Brasília, DF, Brazil

**Burcu Dincgez Cakmak,** University of Health Sciences Turkey, İstanbul, Turkey

**Caetano Petrini,** Universidade de Uberaba, Uberaba, MG, Brazil

**Caio Hartman,** Universidade Estadual de Campinas, Campinas, SP, Brazil

**Caio Lessa,** Universidade de São Paulo, Ribeirão Preto, SP, Brazil

**Caio Prado,** Universidade de São Paulo, Ribeirão Preto, SP, Brazil

**Camila Vasconcelos,** Universidade Federal do Ceara, Fortaleza, CE, Brazil

**Carla Andreucci,** Universidade Federal de São Carlos, São Carlos, SP, Brazil

**Carla Betina Andreucci Polido,** Universidade Federal de São Carlos, São Carlos, SP, Brazil

**Carla Carvalho,** Universidade Estadual de Campinas, Campinas, SP, Brazil

**Carla Nonino,** Universidade de São Paulo, Ribeirão Preto, SP, Brazil

**Carla Piccinato,** Universidade de São Paulo, Ribeirão Preto, SP, Brazil

**Carla Vanin,** Irmandade Complexo Santa Casa de Misericórdia de Porto Alegre, Porto Alegre, RS, Brazil

**Carla Vitola,** Universidade Federal do Rio Grande, Rio Grande, RS, Brazil

**Carlos Alberto Petta,** Hospital Sírio Libanês, São Paulo, SP, Brazil

**Carlos Andrade,** Fundação Oswaldo Cruz, Rio de Janeiro, RJ, Brazil

**Carlos Dias Júnior,** Universidade Estadual Paulista “Júlio de Mesquita Filho”, Botucatu, SP, Brazil

**Carlos Eduardo Poli-de-Figueiredo,** Pontificia Universidade Catolica do Rio Grande do Sul, Porto Alegre, RS, Brazil

**Carlos Henrique Silva,** Hospital Mater Dei, Belo Horizonte, MG, Brazil

**Carlos Maganha,** Faculdade de Ciências Médicas de São José dos Campos, São José dos Campos, SP, Brazil

**Carlos Manuel Ortiz Mendoza,** Centro Medico Dalinde, Cuauhtémoc, México

**Carlos Petta,** Faculdade Ciências Médicas de Minas Gerais, Belo Horizonte, MG, Brazil

**Carlos Politano,** Universidade Estadual de Campinas, Campinas, SP, Brazil

**Carolina Amaral Moreira,** Universidade Estadual de Campinas, Campinas, SP, Brazil

**Carolina Oderich,** Universidade Federal do Rio Grande do Sul, Porto Alegre, RS, Brazil

**Carolina Sales Vieira,** Universidade de São Paulo, Ribeirão Preto, SP, Brazil

**Carolina Valle,** Universidade Estadual de Campinas, Campinas, SP, Brazil

**Caroline Mantavani,** Universidade de São Paulo, São Paulo, SP, Brazil

**Cassia Juliato,** Universidade Estadual de Campinas, Campinas, SP, Brazil

**Cassia Raquel Teatin Juliato,** Universidade Estadual de Campinas, Campinas, SP, Brazil

**Catarna Horta,** Universidade de São Paulo, Ribeirão Preto, SP, Brazil

**Catherine Yzydorczyk,** Centre Hospitalier Universitaire Vaudois, Lausanne, Vaud, Suisse

**Cecília Caetano,** Bayer, São Paulo, SP, Brazil

**Cecília Roteli Martins,** Faculdade de Medicina do ABC, Santo André, SP, Brazil

**Cheung,** R. Y. K. , The Chinese University of Hong Kong, Hong Kong

**Christianne Lok,** The Netherlands Cancer Institute, Amsterdam, Netherlands

**Chung Man Chin,** Universidade Estadual Paulista “Júlio de Mesquita Filho”, Botucatu, SP, Brazil

**Cícero Urban,** Hospital Nossa Senhora das Graças, São Paulo, SP, Brazil

**Cinthia Cazarin,** Universidade Estadual de Campinas, Campinas, SP, Brazil

**Cirbia Campos,** Universidade Estadual de Campinas, Campinas, SP, Brazil

**Claudia Hanson,** London School of Hygiene & Tropical Medicine, London, UK

**Cláudia Jacyntho,** Centro de Prevenção de Câncer Anogenital Serviços Médicos, São Paulo, SP, Brazil

**Cláudia Lodi,** Faculdade de Ciencias Medicas de Minas Gerais, Belo Horizonte, MG, Brazil

**Cláudia Lourdes Soares Laranjeira,** Hospital Mater Dei, Belo Horizonte, MG, Brazil

**Claudia Saunders,** Universidade Federal do Rio de Janeiro, Rio de Janeiro, RJ, Brazil

**Claudio Emilio Bonduki,** Universidade Federal de São Paulo, São Paulo, SP, Brazil

**Claudio Gil Soares de Araujo,** Clínica de Medicina do Exercício, Rio de Janeiro, RJ, Brazil

**Claudio Lera Orsatti,** Unoeste, Presidente Prudente, SP, Brazil

**Conceição Simões,** Hospital de Base Dr. Ary Pinheiro, Porto Velho, RO, Brazil

**Conrado Milani Coutinho,** Universidade de São Paulo, Ribeirão Preto, SP, Brazil

**Conrado Ragazini,** Universidade de São Paulo, Ribeirão Preto, SP, Brazil

**Coskun Umit,** Ankara Ataturk Sanatorium Training and Research Hospital, Ankara, Turkey

**Cristiane de Freitas Paganoti,** Universidade de São Paulo, São Paulo, SP, Brazil

**Cristiani Carboni Fornari,** Faculdade Inspirar, São Paulo, SP, Brazil

**Cristiano Bertolossi Marta,** Universidade Estadual de Campinas, Campinas, SP, Brazil

**Cristiano Salazar,** Universidade Federal do Rio Grande do Sul, Porto Alegre, RS, Brazil

**Cristina Anton,** Universidade de São Paulo, São Paulo, SP, Brazil

**Cristina Benetti-Pinto,** Universidade Estadual de Campinas, Campinas, SP, Brazil

**Cristina Guazelli,** Universidade Federal de São Paulo, São Paulo, SP, Brazil

**Cristina Laguna Benetti Pinto,** Universidade Estadual de Campinas, Campinas, SP, Brazil

**Cristina Silva José,** Universidad Politecnica de Madrid, Madrid, Espanha

**Cristine Ferreira,** Universidade de São Paulo, Ribeirão Preto, SP, Brazil

**Cristine Konopka,** Universidade Federal de Santa Maria, Santa Maria, RS, Brazil

**Cynthia Dela Cruz,** University of Colorado Boulder, Colorado, USA

**Dagmar Wertaschnigg,** Monash University, Melbourne, Victoria, Australia

**Daiane Gottlieb,** Santa Casa de Misericórdia de Porto Alegre, Porto Alegre, RS, Brazil

**Daiane Sofia Paulino,** Universidade Estadual de Campinas, Campinas, SP, Brazil

**Dani Ejzenberg,** Universidade de São Paulo, São Paulo, SP, Brazil

**Daniel Dias,** Universidade Estadual Paulista “Júlio de Mesquita Filho”, Botucatu, SP, Brazil

**Daniel Dias Spadoto,** Universidade Estadual Paulista Júlio de Mesquita Filho, Botucatu, SP, Brazil

**Daniel Fatori,** Universidade São Paulo, São Paulo, SP, Brazil

**Daniel Guimarães Tiezzi,** Universidade de São Paulo, Ribeirão Preto, SP, Brazil

**Daniel Rolnik,** Monash Institute of Medical Research Centre for Cancer Research, Obstetrics and Gynaecology, Monash, Austrália

**Daniel Spadoto Dias,** Universidade Estadual Paulista “Júlio de Mesquita Filho”, Botucatu, SP, Brazil

**Daniel Tiezzi,** Universidade de São Paulo, Ribeirão Preto, SP, Brazil

**Daniela Angerame Yela Gomes,** Universidade Estadual de Campinas, Campinas, SP, Brazil

**Daniela Prado,** Universidade Federal de Sergipe, São Cristovão, SE, Brazil

**Daniela Sartorelli,** Universidade de São Paulo, Ribeirão Preto, SP, Brazil

**Daniela Vettori,** Universidade Federal do Rio Grande do Sul, Porto Alegre, RS, Brazil

**Daniela Yela,** Universidade Estadual de Campinas, Campinas, SP, Brazil

**Danieli Andrade,** Universidade de São Paulo, São Paulo, SP, Brazil

**Danilo Buca,** G d’Annunzio University of Chieti-Pescara, Chieti, Italy

**Danilo Pastore,** Universidade Estadual de Campinas, Campinas, SP, Brazil

**Dartagnan Pinto Guedes,** Universidade Estadual de Londrina, Londrina, PR, Brazil

**David Barreira Gomes Sobrinho,** Universidade Estadual Paulista Julio de Mesquita Filho, Botucatu, SP, Brazil

**Débora Britto,** Universidade Federal do Ceará, Fortaleza, CE, Brazil

**Debora Faria-Schützer,** Universidade Estadual de Campinas, Campinas, SP, Brazil

**Debora Farias Leite,** Universidade Federal de Pernambuco, Recife, PE, Brazil

**Debora Leite,** Universidade Federal de Pernambuco, Recife, PE, Brazil

**Delio Conde,** Hospital Materno Infantil, Goiânia, GO, Brazil

**Delio Marques Conde,** Hospital for Maternal and Child Healthcare, Goiânia, GO, Brazil

**Delzio Bicalho,** Clínica Delzio Bicalho, Belo Horizonte, MG, Brazil

**Denis Nascimento,** Universidade Federal do Parana, Curitiba, PR, Brazil

**Denise Cartagena,** Universidade de São Paulo, Ribeirão Preto, SP, Brazil

**Denise Maia,** Universidade do Estado do Rio de Janeiro, Rio de Janeiro, RJ, Brazil

**Denisse Cartagena-Ramos,** Universidade de São Paulo, Ribeirão Preto, SP, Brazil

**Diama Bhadra Andrade Peixoto do Vale,** Universidade Estadual de Campinas, Campinas, SP, Brazil

**Diama Vale,** Universidade Estadual de Campinas, Campinas, SP, Brazil

**Diesa Pinheiro,** Universidade Federal de Ciencias da Saude de Porto Alegre, Porto Alegre, RS, Brazil

**Dietmar Schlembach,** VIivantes Klinikum Neukolln, Berlin, Alemanha

**Donald Nelson,** Washington University in St Louis, St Louis, Estados Unidos

**Eddie Fernando Candido Murta,** Universidade Federal do Triângulo Mineiro, Uberaba, MG, Brazil

**Edilberto Rocha Filoh,** Universidade Federal de Pernambuco, Recife, PE, Brazil

**Edimárlei Valério,** Hospital de Clinicas de Porto Alegre, Porto Alegre, RS, Brazil

**Edison Capp,** Universidade Federal do Rio Grande do Sul, Porto Alegre, RS, Brazil

**Edna Gondim,** Universidade Federal do Ceara, Fortaleza, CE, Brazil

**Edson Borges Junior,** Fertility Medical Group, São Paulo, SP, Brazil

**Edson Cunha Filho,** Pontifícia Universidade Católica do Rio Grande do Sul, Porto Alegre, RS, Brazil

**Edson Cunha Filho,** Pontificia Universidade Catolica do Rio Grande do Sul, Porto Alegre, RS, Brazil

**Edson Ferreira-Filho,** Universidade de São Paulo, São Paulo, SP, Brazil

**Edson Pereira-Fiho,** Universidade de São Paulo, São Paulo, SP, Brazil

**Edson Rudey,** Universidade Federal de Santa Catarina, Florianópolis, SC, Brazil

**Edson Vieira Cunha,** Pontificia Universidade Catolica do Rio Grande do Sul, Porto Alegre, RS, Brazil

**Edson Zangiacomi Martinez,** Universidade de São Paulo, Ribeirão Preto, SP, Brazil

**Eduardo Becker,** Universidade Federal do Rio Grande do Sul, Porto Alegre, RS, Brazil

**Eduardo Cândido,** Universidade Federal de Minas Gerais, Belo Horizonte, MG, Brazil

**Eduardo Cordioli,** Hospital Israelita Albert Einstein, São Paulo, SP, Brazil

**Eduardo Fernandes,** Universidade Federal de Minas Gerais, Belo Horizonte, MG, Brazil

**Eduardo Madeira,** Universidade Estadual do Piaui, Floriano, PI, Brazil

**Eduardo Millen,** Leblon Medical Center, Lebon, RJ, Brazil

**Eduardo Passos,** Universidade Federal do Rio Grande do Sul, Porto Alegre, RS, Brazil

**Eduardo Paulino,** Instituto Nacional de Cancer, São Paulo, SP, Brazil

**Eduardo Pessoa,** Universidade Estadual Paulista “Júlio de Mesquita Filho”, Botucatu, SP, Brazil

**Eduardo Schor,** Universidade Federal de São Paulo, São Paulo, SP, Brazil

**Eduardo Souza,** Universidade Federal de São Paulo, São Paulo, SP, Brazil

**Eduardo Vieira,** Universidade de São Paulo, São Paulo, SP, Brazil

**Edward Araújo Júnior,** Universidade Federal de São Paulo, São Paulo, SP, Brazil

**Egle Couto,** Universidade Estadual de Campinas, Campinas, SP, Brazil

**Elaine Barros Ferreira,** Universidade de Brasilia, Brasília, DF, Brazil

**Elaine Candido,** Universidade Estadual de Campinas, Campinas, SP, Brazil

**Elaine Guirro,** Universidade de São Paulo, Ribeirão Preto, SP, Brazil

**Elisabeth Meloni,** Universidade de São Paulo, Ribeirão Preto, SP, Brazil

**Elsa Aida Gay de Pereyra,** Universidade de São Paulo, São Paulo, SP, Brazil

**Elton Ferreira,** Universidade Estadual de Campinas, Campinas, SP, Brazil

**Elyonara Figueiredo,** Universidade Federal de Minas Gerais, Belo Horizonte, MG, Brazil

**Elza Uberti,** Santa Casa de Misericórdia de Porto Alegre, Porto Alegre, RS, Brazil

**Emanoela Toledo,** Universidade de São Paulo, Ribeirão Preto, SP, Brazil

**Emerson Oliveira,** Faculdade de Medicina do ABC, Santo André, SP, Brazil

**Emine Aydin,** Hacettepe Universitesi Tip Fakultesi, Ankara, Turkey

**Emmanuele Janini,** University of Rome, Roma, Italy

**Emmeline Lee Daniel Spadoto Dias,** Universidade Estadual Paulista “Júlio de Mesquita Filho”, Botucatu, SP, Brazil

**Ênio Damaso,** Universidade de São Paulo, Ribeirão Preto, SP, Brazil

**Enoch Barreto,** Hospital e Maternidade Vila Nova Cachoerinha, São Paulo, SP, Brazil

**Ernesto Figueiró,** University of Toronto, Toronto, Canadá

**ERSİN ÇİNTESUN,** Selcuk Universitesi Tip Fakultesi, Selçuklu/Konya, Turkey

**Etelvino de Souza Trindade,** Instituto Verhum, Brasília, DF, Brazil

**Eura Lage,** Universidade Federal de Minas Gerais, Blo Horizonte, MG, Brazil

**Evanguelia dos Santos,** Universidade Federal de Santa Catarina, Florianópolis, SC, Brazil

**Evelyn Traina,** Universidade Federal de São Paulo, São Paulo, SP, Brazil

**Fabiane Vale,** Universidade Federal de Minas Gerais, Belo Horizonte, MG, Brazil

**Fabio Bagnoli,** Faculdade de Ciências Médicas da Santa Casa de São Paulo, São Paulo, SP, Brazil

**Fabio Cabar,** Universidade de São Paulo, São Paulo, SP, Brazil

**Fabio Comim,** Universidade Federal de Minas Gerais, Belo Horizonte, MG, Brazil

**Fabio Moraes,** Universidade Federal do Tocantins, Palmas, TO, Brazil

**Fabio Ohara,** Faculdade de Ciencias Medicas da Santa Casa de Sao Paulo, São Paulo, SP, Brazil

**Fabio Orsatti,** Universidade Federal do Triângulo Mineiro, Uberaba, MG, Brazil

**Fabio Vasconcellos Comim,** Universidade Federal de Minas Gerais, Belo Horizonte, MG, Brazil

**Fabrício Brenelli,** Universidade Estadual de Campinas, Campinas, SP, Brazil

**Fanny Corrlaes Rios,** Instituto de Prevision Social, Asunción, Paraguay

**Fatemeh Darsareh,** Hormozgan University of Medical Sciences, Bandarabbas, Iran

**Fatima Fitz,** Universidade Federal de São Paulo, São Paulo, SP, Brazil

**Fatima Penso,** Maternidade Leila Diniz, Rio de Janeiro, RJ, Brazil

**Felipe Zerwes,** Pontificia Universidade Católica do Rio Grande do Sul, Porto Alegre, RS, Brazil

**Fernanda Campos,** Universidade Federal do Estado do Rio de Janeiro, Rio de Janeiro, RJ, Brazil

**Fernanda Dapper Machado,** Universidade Federal do Rio Grande do Sul, Porto Alegre, RS, Brazil

**Fernanda Garanhani de Castro Surita,** Universidade Estadual de Campinas, Campinas, SP, Brazil

**Fernanda Kesselring Tso,** Universidade Federal de São Paulo, São Paulo, SP, Brazil

**Fernanda Tso,** Universidade Federal de São Paulo, São Paulo, SP, Brazil

**Fernando Bastos,** Universidade Federal de Minas Gerais, Belo Horizonte, MG, Brazil

**Fernando Gomes,** Universidade de São Paulo, Ribeirão Preto, SP, Brazil

**Fernando Maia Peixoto-Filho,** Fundacao Oswaldo Cruz, Brasilía, DF, Brazil

**Fernando Marcos dos Reis,** Universidade Federal de Minas Gerais, Belo Horizonte, MG, Brazil

**Fernando Marcos Gomes,** Universidade de São Paulo, Ribeirão Preto, SP, Brazil

**Fernando Valério,** Universidade de São Paulo, Ribeirão Preto, SP, Brazil

**Flavia Bueloni-Dias,** Universidade Estadual Paulista “Júlio de Mesquita Filho”, Botucatu, SP, Brazil

**Flávia Fairbanks Lima de Oliveira Marino,** Hospital das Clínicas, Faculdade de Medicina, Universidade de São Paulo, São Paulo, SP, Brazil

**Flavia Gomes Sponholz,** Universidade de São Paulo, São Paulo, SP, Brazil

**Flávia Sponholz,** Universidade de São Paulo, São Paulo, SP, Brazil

**Florence Robert-Gangneux,** Université de Rennes, Rennes, French

**Florentino Mendes,** Universidade Federal de Ciências da Saúde de Porto Alegre, Porto Alegre, RS, Brazil

**Francisco Cavalcante,** Hospital Geral de Fortaleza, Fortaleza, CE, Brazil

**Francisco José Cândido dos Reis,** Universidade de São Paulo, Ribeirão Preto, SP, Brazil

**Francisco Medeiros,** Universidade Federal do Ceara, Fortaleza, CE, Brazil

**Francisco Pereira,** Faculdade Ciências Médicas de Minas Gerais, Belo Horizonte, MG, Brazil

**Francisco Prota,** Universidade Federal do Rio Grande do Sul, Porto Alegre, RS, Brazil

**Francisco Ramos Filho,** Santa Casa de Misericórdia de Belo Horizonte, Belo Horizonte, MG, Brazil

**Francisco Reis,** Universidade de São Paulo, Ribeirão Preto, SP, Brazil

**Francisco Souza,** Centro Universitário Lusíada, Santos, SP, Brazil

**Franklin Pimentel,** Universidade de São Paulo, São Paulo, SP, Brazil

**Frederico Correa,** Universidade de Brasilia, Brasilia, DF, Brazil

**Gabriel Aleixo,** Universidade do Oeste Paulista, Guarujá, SP, Brazil

**Gabriel Costa Osanan,** Universidade Federal de Minas Gerais, Belo Horizonte, MG, Brazil

**Gabriel Veber,** Universidade Federal do Rio Grande do Sul, Porto Alegre, RS, Brazil

**Gabriela Paiva,** Universidade Federal do Rio de Janeiro, RJ, Brazil

**Gabriela Rezende,** Universidade Estadual de Campinas, Campinas, SP, Brazil

**Gabriele Tonni,** AUSL Reggio Emilia, Reggio Emilia, Italy

**Gajanan Bhat,** University of Georgia, GA, USA

**George SH Yeo,** KK Women’s and Children’s Hospital, Singapore

**Geraldine Giraudet,** University Hospital of Lille, France

**Geraldo Duarte,** Universidade de São Paulo, Ribeirão Preto, SP, Brazil

**Gerson Cláudio Crott,** Universidade de São Paulo, Ribeirão Preto, SP, Brazil

**Gianluca Montarani Vergallo,** University of Rome La Sapienza, Roma, Italy

**Gianpaollo Grisolia,** Università degli Studi di Milano, Milão, Italy

**Gilberto Kac,** Universidade Federal do Rio de Janeiro, Rio de Janeiro, RJ, Brazil

**Giordana Braga,** Universidade de São Paulo, Ribeirão Preto, SP, Brazil

**Giorgina Piccoli,** Centre Hospitalier Le Mans, Le Mans, França

**Giorgio Tondello,** Maternidade Darcy Vargas, Joinville, SC, Brazil

**Gisele Breda,** Santa Casa de Misericórdia de Porto Alegre, Porto Alegre, RS, Brazil

**Gisele Gribel,** Universidade Federal do Rio de Janeiro, Rio de Janeiro, RJ, Brazil

**Gislaine Kogure,** Universidade de São Paulo, Ribeirão Preto, SP, Brazil

**Giuliane Lajes,** Universidade Estadual de Campinas, Campinas, SP, Brazil

**Giuliano Bedoschi,** Universidade de São Paulo, Ribeirão Preto, SP, Brazil

**Giuliano Duarte,** Universidade Estadual de Campinas, Campinas, SP, Brazil

**Giuliano Gardenghi,** Hospital Encore, Goiânia, GO, Brazil

**Giuliano Tosello,** Universidade do Oeste Paulista, Presidente Prudente, SP, Brazil

**Giuseppe Rizzo,** Universita degli Studi di Roma Tor Vergata, Roma, Italy

**Glaucia Pereira,** Universidade Estadual de Campinas, Campinas, SP, Brazil

**Glaucia Varella,** Universidade Estadual de Campinas, Campinas, SP, Brazil

**Glauco Baiocchi,** A.C.Camargo Cancer Center, São Paulo, SP, Brazil

**Gokcen Orgul,** Hacettepe Üniversitesi Tıp Fakültesi, Ankara, Turkey

**Guilherme Fernandes,** Faculdade de Medicina do ABC, Santo André, SP, Brazil

**Guilherme Garcia,** Hospital Paulistano, São Paulo, SP, Brazil

**Guilherme Jesus,** Universidade do Estado do Rio de Janeiro, Rio de Janeiro, RJ, Brazil

**Guilherme Nobrega,** Universidade Estadual de Campinas, Campinas, SP, Brazil

**Guilherme Rezende,** Faculdade da Saúde e Ecologia Humana, Vespasiano, MG, Brazil

**Guilherme Zanluchi,** Beneficência Portuguesa de São Paulo, São Paulo, SP, Brazil

**Gustavo Antônio de Souza,** Universidade Estadual de Campinas, Campinas, SP, Brazil

**Gustavo Focchi,** Universidade Federal de São Paulo, São Paulo, SP, Brazil

**Gustavo Lourenço,** Universidade Estadual de Campinas, Campinas, SP, Brazil

**Gustavo Meirelles,** Universidade Federal de Ouro Preto, Ouro Preto, MG, Brazil

**Gustavo Pileggi-Castro,** Universidade de São Paulo, São Paulo, SP, Brazil

**Gustavo Soares,** Universidade Federal do Rio Grande do Norte, Natal, RN, Brazil

**Hasan Inal,** Konya Training and Research Hospital, Belgium

**Helaine Milanez,** Universidade Estadual de Campinas, Campinas, SP, Brazil

**Helen L Del Puerto,** Universidade Federal de Minas Gerais, Belo Horizonte, MG, Brazil

**Helena Hachul,** Hospital Santa Marcelina, São Paulo, SP, Brazil

**Helena Malvezzi,** Hospital Israelita Albert Einstein, São Paulo, SP, Brazil

**Helena Schmid,** Universidade Federal do Rio Grande do Sul, Porto Alegre, RS, Brazil

**Helena Slongo,** Universidade Federal do Paraná, Curitiba, PR, Brazil

**Helizabet Ribeiro,** Faculdade de Ciencias Medicas da Santa Casa de São Paulo, São Paulo, SP, Brazil

**Helmer Herren,** Universidade de São Paulo, Ribeirão Preto, SP, Brazil

**Heloisa Bettiol,** Universidade de São Paulo, Ribeirão Preto, SP, Brazil

**Heloisa Carvalho,** Universidade de São Paulo, São Paulo, SP, Brazil

**Henk Schonewille,** University of Amsterdam, Amsterdã

**Henri Korkes,** Pontificia Universidade Catolica de São Paulo, São Paulo, SP, Brazil

**Heron Werner,** Clinica de Diagnostico por Imagem, Botafogo, RJ, Brazil

**Heverton Amorim,** Universidade Federal do Rio Grande do Sul, Porto Alegre, RS, Brazil

**Hilka Alves Pereira,** Universidade Federal do Amazonas, Manaus, AM, Brazil

**Hirokazu Usui,** Chiba University, Chiba, Japão

**Homero Guidi,** Universidade de São Paulo, São Paulo, SP, Brazil

**Humberto Hirakawa,** Universidade Federal de São Carlos, São Carlos, SP, Brazil

**Iara Linhares,** Universidade de São Paulo, São Paulo, SP, Brazil

**Ibrahim Abdelazim,** Kuwait Oil Company, Kuwait

**Ilza Maria Urbano Monteiro,** Universidade Estadual de Campinas, Campinas, SP, Brazil

**Ines Carvalho,** Universidade Federal de Minas Gerais, Belo Horizonte, MG, Brazil

**Inessa Beraldo de Andrade Bonomi,** Universidade José do Rosário Vellano, Alfenas, MG, Brazil

**Ingrid Britto,** Faculdade de Ciencias Medicas da Santa Casa de Sao Paulo, São Paulo, SP, Brazil

**Ionara Barcelos,** Universidade Estadual do Oeste do Parana, Cascavel, PR, Brazil

**Iracema Calderon,** Universidade Estadual Paulista Julio de Mesquita Filho, Botucatu, SP, Brazil

**Isabel Cristina Esposito Sopreso,** Universidade de São Paulo, São Paulo, SP, Brazil

**Isabel Esposito Sorpreso,** Universidade de São Paulo, São Paulo, SP, Brazil

**Isabel Guimarães,** Universidade Federal Fluminense, Niterói, RJ, Brazil

**Isabel Maria Correia,** Universidade Federal de Minas Gerais, Belo Horizonte, MG, Brazil

**Isabella Parente,** Sociedade de Assistencia a Maternidade Escola Assis Chateaubriand, Fortaleza, CE, Brazil

**Ivaldo Silva,** Universidade Federal de São Paulo, São Paulo, SP, Brazil

**Ivan Montenegro,** Hospital de Clinicas de Porto Alegre, Porto Alegre, RS, Brazil

**Ivan Penna,** Universidade Federal Fluminense, Niteroi, RJ, Brazil

**Ivana Babovic,** University of Belgrade, Belgrade, Sérvia

**Izildinha Maestá,** Universidade Estadual Paulista Júlio de Mesquita Filho, São paulo, SP, Brazil

**Jader Cruz,** Centro Hospitalar de Lisboa, Lisboa, Portugal

**Jaime Kulak Junior,** Universidade Federal do Parana, Curitiba, PR, Brazil

**Jan Filip Baekelandt,** Imelda Hosp Bonheiden, Belgium

**Jan Pawel Pachnicki,** Universidade Federal do Paraná, Curtiba, PR, Brazil

**Janaina Cristina Crispim,** Universidade Federal do Rio Grande do Norte, Natal, RN, Brazil

**Janaina Senra,** Universidade Federal de Minas Gerais, Belo Horizonte, MG, Brazil

**Janete Vettorazzi,** Universidade Federal do Rio Grande do Sul, Porto Alegre, RS, Brazil

**Janice Tartari,** Universidade Federal de Ciências da Saúde de Porto Alegre, RS, Brazil

**Jaqueline Neves Lubianca,** Hospital de Clínicas de Porto Alegre, Porto Alegre, RS, Brazil

**Jarbas Magalhães,** Universidade Estadual de Campinas, Campinas, SP, Brazil

**Javier Miguelez,** Grupo Fleury, São Paulo, SP, Brazil

**Jayshree Ramkrishna,** Monash University, Melbourne, Victoria, Australia

**Jean Carl Silva,** Universidade da Região de Joinville, Joinville, SC, Brazil

**Jennifer Chipps,** University of the Western Cape, Cape Town, South Africa

**Jessica Zandona,** Universidade de Passo Fundo, Passo Fundo, RS, Brazil

**Jesus Paula Carvalho,** Universidade de São Paulo, São Paulo, SP, Brazil

**Jittima Manonai,** Ramathibodi Medical Journal Office, Bangkok, Tailândia

**João Bennini,** Universidade Estadual de Campinas, Campinas, SP, Brazil

**João Bosco,** Faculdade de Medicina de Jundiai, Jundiaí, SP, Brazil

**João Neto,** Universidade Federal do Maranhão, São Luís, MA, Brazil

**João Renato Benini Júnior,** Universidade Estadual de Campinas, Campinas, SP, Brazil

**João Sabino Cunha Filho,** Universidade Federal do Rio Grande do Sul, Porto Alegre, RS, Brazil

**Joelcio Abbade,** Universidade Estadual Paulista “Júlio de Mesquita Filho”, São Paulo, SP, Brazil

**Joelma Queiroz,** Universidade de São Paulo, São Paulo, SP, Brazil

**Johannes Duvekot,** Erasmus Universiteit Rotterdam, Rotterdam, Holanda

**Jorge Leão,** Universidade do Estado do Amazonas, Manaus, AM, Brazil

**Jorge Rezende Filho,** Universidade Federal do Rio de Janeiro, Rio de Janeiro, RJ, Brazil

**José Ananias Vasconcelos Neto,** Hospital Geral de Fortaleza, Fortaleza, CE, Brazil

**José Antônio Magalhães,** Universidade Federal do Rio Grande do Sul, Porto Alegre, RS, Brazil

**José Antônio Rojas-Suarez,** Universidad de Cartagena, Bolivar, Colombia

**José Barreto Campello Carvalheira,** Universidade Estadual de Campinas, Campinas, SP, Brazil

**Jose Candido Xavier,** Instituto de Patologia de Araçatuba, Araçatuba, SP, Brazil

**José Carlos Peraçoli,** Universidade Estadual Paulista Julio de Mesquita Filho, Botucatu, SP, Brazil

**José Carlos Sadalla,** Universidade de São Paulo, São Paulo, SP, Brazil

**José Deus,** Universidade Federal de Goias, Goiania, GO, Brazil

**José Eleutério Júnior,** Universidade Federal do Ceara, Fortaleza, CE, Brazil

**José Geraldo Lopes Ramos,** Universidade Federal do Rio Grande do Sul, Porto Alegre, RS, Brazil

**José Guilherme Cecatti,** Universidade Estadual de Campinas, Campinas, SP, Brazil

**José Maria Soares Júnior,** Universidade de São Paulo, São Paulo, SP, Brazil

**José Mauro Madi,** Universidade de Caxias do Sul, Caxias do Sul, RS, Brazil

**Jose Paulo Guida,** Universidade Estadual de Campinas, Campinas, SP, Brazil

**José Peraçoli,** Universidade Estadual Paulista “Júlio de Mesquita Filho”, Botucatu, SP, Brazil

**José Ramos,** Universidade Federal do Rio Grande do Sul, Porto Alegre, RS, Brazil

**Jose Rojas-Suarez,** Universidad de Cartagena, Bolívar, Colombia

**José Tadeu Martins,** Universidade Federal de São Paulo, São Paulo, SP, Brazil

**José Wictor Borges,** Universidade Federal do Piaui, Floriano, PI, Brazil

**José Zanardi,** Universidade de São Paulo, São Paulo, SP, Brazil

**Juan Blumel,** Universidade Estadual de Campinas, Campinas, SP, Brazil

**Juan Luis Alcazar,** Clinica Universidad de Navarra, Navarra, Espanha

**Juin Yee Kong,** KK Women’s and Children’s Hospital, Kampong Java, Singapura

**Julia C. Gage,** National Institute of Health, Nova Iorque, USA

**Julia Troncon,** Universidade de São Paulo, Ribeirão Preto, SP, Brazil

**Juliana Barra,** Universidade Federal de Minas Gerais, Belo Horizonte, MG, Brazil

**Juliana Mello,** Universidade Federal do Rio Grande do Sul, Porto Alegre, RS, Brazil

**Juliana Meola,** Universidade de São Paulo, Ribeirão Preto, SP, Brazil

**Juliana Poli,** Universidade de Toroto, Toronto, Canada

**Juliana Vaz,** Universidade Federal de Pelotas, Pelotas, RS, Brazil

**Juliana Yoko,** Universidade Estadual de Campinas, Campinas, SP, Brazil

**Julio Cesar Rosa e Silva,** Universidade de São Paulo, Ribeirão Preto, SP, Brazil

**Julio Cesar Teixeira,** Universidade Estadual de Campinas, Campinas, SP, Brazil

**Julio Elito Júnior,** Universidade Federal de São Paulo, São Paulo, SP, Brazil

**Julio Geminiani,** Pontifícia Universidade Católica de São Paulo, São Paulo, SP, Brazil

**Julisa Ribalta,** Universidade Federal de São Paulo, São Paulo, SP, Brazil

**Jussara Mayrink,** Universidade do Estado de Minas Gerais, Belo Horizonte, MG, Brazil

**Kaled Mikki Zimmo,** Oslo University Hospital, Rikshospitalet, Oslo, Noruega

**Karayna Fernandes,** Faculdade de Medicina de Jundiaí, Jundiaí, SP, Brazil

**Karen Abrão,** Centro Universitário FACENS, Sorocaba, SP, Brazil

**Karime Kalil,** Johns Hopkins University Bloomberg School of Public Health, Estados Unidos

**Karina de Cássia Ribeiro,** Faculdade de Ciências Médicas da Santa Casa de São Paulo, São Paulo, SP, Brazil

**Karina Lopes,** Instituto de Medicina Integral Professor Fernando Figueira, Recife, PE, Brazil

**Karina Pontes,** Universidade Federal de São Paulo, São Paulo, SP, Brazil

**Karina Rezende,** Universidade Federal do Rio de Janeiro, Rio de Janeiro, RJ, Brazil

**Kassam Mahomed,** Queensland Health: Brisbane, QLD, AU

**Katia Borgia Barbosa Pagnano,** Universidade Estadual de Campinas, Campinas, SP, Brazil

**Kelly Pereira Coca,** Universidade Federal de São Paulo, São Paulo, SP, Brazil

**Kerac Falk,** Emory University School of Medicine, Atlanta, USA

**Laise Veoloso,** Universidade Estadual do Piauí, Floriano, PI, Brazil

**Lara Termini,** Universidade de São Paulo, São Paulo, SP, Brazil

**Larissa Coutinho,** Universidade Federal de Juiz de Fora, Juiz de Fora, MG, Brazil

**Larissa Eloy,** Universidade Estadual de Campinas, Campinas, SP, Brazil

**Larissa Mandarano,** Universidade de São Paulo, Ribeirão Preto, SP, Brazil

**Larissa Rodrigues,** Universidade Estadual de Campinas, Campinas, SP, Brazil

**Laura Costa,** Universidade de Pernambuco, Recife, PE, Brazil

**Laura Zaiden e Ferreira Pinto,** Universidade Federal do Rio de Janeiro, Rio de Janeiro, RJ, Brazil

**Lawrence Hsu Lin,** Universidade de São Paulo, São Paulo, SP, Brazil

**Lawrence Lin,** NYU Langone Health, New York, USA

**Leandro Oliveira,** Universidade Estadual Paulista Júlio de Mesquita Filho, Botucatu, SP, Brazil

**Leandro Resende,** Hospital de Base do Distrito Federal, Brasília, DF, Brazil

**Leila Katz,** Instituto de Medicina Integral Professor Fernando Figueira, Recife, PE, Brazil

**Lenita Zajdenverg,** Universidade Federal do Rio de Janeiro, Rio de Janeiro, RJ, Brazil

**Leonardo Bezerra,** Universidade Federal do Ceara, Fortaleza, CE, Brazil

**Leonardo da Silva,** Universidade Estadual de Campinas, Campinas, SP, Brazil

**Leonardo Orlandini,** Universidade de São Paulo, Ribeirão Preto, SP, Brazil

**Leopoldo de Oliveira Tso,** Faculdade de Ciências Médicas da Santa Casa de São Paulo,

**Leticia Arruda,** Universidade Federal do Rio Grande do Sul, Porto Alegre, RS, Brazil

**Leticia Katz,** Secretaria Estadual de Saúde de Pernambuco, Recife, PE, Brazil

**Leticia Viçosa Pires,** Universidade Federal de Ciências da Saúde de Porto Alegre, Porto Alegre, RS, Brazil

**Li Dai,** Sichuan University, Si Chuan Sheng, China

**Lia Cruz Vaz da Costa Damásio,** Universidade Federal do Piauí, Teresina, PI, Brazil

**Lia Damásio,** Universidade Federal do Piaui, Teresina, PI, Brazil

**Licia Santana,** Universidade de São Paulo, Ribeirão Preto, SP, Brazil

**Lilian Hsu,** Faculdade de Ciências Médicas da Santa Casa de São Paulo, São Paulo, SP, Brazil

**Liliane Andrade,** Universidade Estadual de Campinas, Campinas, SP, Brazil

**Linei Urban,** Universidade Federal do Rio Grande do Sul, Porto Alegre, RS, Brazil

**Lorena Urbanetz,** Universidade de São Paulo, São Paulo, SP, Brazil

**Louise De Brot,** A.C.Camargo Cancer Center, São Paulo, SP, Brazil

**Lucas Almeida das Chagas,** Universidade Federal de São Paulo, São Paulo, SP, Brazil

**Lucas Bezerra,** Centro Universitario Mauricio de Nassau, Aracaju, SE, Brazil

**Lucas Schreiner,** Pontificia Universidade Catolica do Rio Grande do Sul, Porto Alegre, RS, Brazil

**Lucas Trigo,** University of Barcelona, Barcelona, Espanha

**Lucia Alves da Silva Lara,** Universidade de São Paulo, Ribeirão Preto, SP, Brazil

**Lucia Helena Simões da Costa Paiva,** Universidade Estadual de Campinas, Campinas, SP, Brazil

**Luciana Brito,** Instituto de Bioética, Direitos Humanos e Gênero, São Paulo, SP, Brazil

**Luciana França,** Hospital Israelita Albert Einstein, São Paulo, SP, Brazil

**Luciana Pietro,** Universidade Paulista, Campinas, SP, Brazil

**Luciana Verçosa,** Universidade Federal do Rio Grande do Sul, Porto Alegre, RS, Brazil

**Luciane Fernandes,** Universidade Federal do Triângulo Mineiro, Uberaba, MG, Brazil

**Luciano Gibran,** Hospital Pérola Byington, São Paulo, SP, Brazil

**Luciano Nardozza,** Universidade Federal de São Paulo, São Paulo, SP, Brazil

**Luciano Pompei,** Faculdade de Medicina do ABC, Santo André, SP, Brazil

**Ludwig Kiesel,** Universitätsklinikum Münster Klinik f.Frauenheilkunde, Münster, Alemanha

**Lufee Wong,** Monash Medical Centre Clayton, Clayton, Australia

**Luis Bahamondes,** Universidade Estadual de Campinas, Campinas, SP, Brazil

**Luis Flavio Gonçalves,** Universidade Federal de São Paulo, São Paulo, SP, Brazil

**Luis Gustado Toledo,** Faculdade de Ciências Médicas da Santa Casa de São Paulo, São Paulo, SP, Brazil

**Luis Jose Catoggio,** Hospital Italiano de Buenos Aires, Buenos Aires, Argentina

**Luisa Guimarães Santos,** Universidade Federal do Rio de Janeiro, Rio de Janeiro, RJ, Brazil

**Luisa Menezes,** Universidade de Pernambuco, Recife, PE, Brazil

**Luisa Villa,** Universidade de São Paulo, São Paulo, SP, Brazil

**Luiz Baccaro,** Universidade Estadual de Campinas, Campinas, SP, Brazil

**Luiz Claudio Thuler,** Instituto Nacional de Cancer, São Paulo, SP, Brazil

**Luiz Flavio Fernandes,** Universidade de São Paulo, São Paulo, SP, Brazil

**Luiz Gustavo Oliveira Brito,** Universidade Estadual de Campinas, Campinas, SP, Brazil

**Luiz Vicente Garcia,** Universidade de São Paulo, Ribeirão Preto, SP, Brazil

**Luiza Aguiar,** Universidade Estadual de Campinas, Campinas, SP, Brazil

**Luiza Riccio,** Universidade de São Paulo, São Paulo, SP, Brazil

**Lynsey Hayward,** Middlemore Hospital, Auckland, Nova Zelândia

**Maciej Słodki,** Institute of Polish Mother’s Health Center, Łódź, Poland

**Mahbobeh Faramarz,** Babol University of Medical Science, Mazandaran Province, Iran

**Maila Neves,** Universidade Federal de Minas Gerais, Belo Horizonte, MG, Brazil

**Maira Alejandra Vera Montoya,** Universidade Federal de Minas Gerais, Belo Horizonte, MG, Brazil

**Maíra Casalechi,** Universidade Federal de Minas Gerais, Belo Horizonte, MG, Brazil

**Maíra Dória,** Hospital Nossa Senhora das Graças, São Paulo, SP, Brazil

**Maira Libertad Takemoto,** Universidade Estadual Paulista, Botucatu, SP, Brazil

**Maira Pinho-Pompeu,** Universidade Estadual de Campinas, Campinas, SP, Brazil

**Maira Rossmann-Machado,** Universidade Estadual de Campinas, Campinas, SP, Brazil

**Malvina Rodrigues,** Universidade Federal do Piauí, Teresina, PI, Brazil

**Manuel Afonso Guimaraes Gonçalves,** Pontifícia Universidade Católica de Campinas, Campinas, SP, Brazil

**Manuel Di Biase,** Univ Illinois, Illinois, Chicago, USA

**Manuel Simões,** Universidade Federal de São Paulo, São Paulo, SP, Brazil

**Marair Sartori,** Universidade Federal de São Paulo, São Paulo, SP, Brazil

**Marcela Coelho Neto,** Universidade de São Paulo, Ribeirão Preto, SP, Brazil

**Marcela Alencar,** Universidade de São Paulo, Ribeirão Preto, SP, Brazil

**Marcela Bardin,** Universidade Estadual de Campinas, Campinas, SP, Brazil

**Marcela Boraschi Marçal,** Faculdade de Medicina de Jundiaí, Jundiaí, SP. Brazil

**Marcela Lacerda,** Hospital Universitário Pedro Ernesto, Rio de Janeiro, RJ, Brazil

**Marcelo Felix da Silva,** Universidade de São Paulo, Ribeirão Preto, SP, Brazil

**Marcelo França,** Universidade Federal de São Paulo, São Paulo, SP, Brazil

**Marcelo Goldani,** Universidade Federal do Rio Grande do Sul, Porto Alegre, RS, Brazil

**Marcelo Luis Nomura,** Universidade Estadual de Campinas, Campinas, SP, Brazil

**Marcelo Marques,** Instituto de Medicina Integral Professor Fernando Figueira, Recife, PE, Brazil

**Marcelo Nomura,** Universidade Estadual de Campinas, Campinas, SP, Brazil

**Marcelo Steiner,** Faculdade de Medicina do ABC, Santo André, SP, Brazil

**Marcia Baldisserotto,** Universidade Federal do Rio de Janeiro, Rio de Janeiro, RJ, Brazil

**Marcia Cardial,** Faculdade de Medicina do ABC, Santo André, SP, Brazil

**Márcia Carneiro,** Universidade Federal de Minas Gerais, Belo Horizonte, MG, Brazil

**Marcia Consolaro,** Universidade Estadual de Maringá, Maringá, PR, Brazil

**Marcia Freitas,** Universidade Federal de Uberlândia, Uberlândia, MG, Brazil

**Marcia Gimenez,** Universidade Federal de São Paulo, São Paulo, SP, Brazil

**Marcia Luiza Binda,** Universidade Federal do Rio Grande do Sul, Porto Alegre, RS, Brazil

**Marcio Alexandre Rodrigues,** Universidade Federal de Minas Gerais, Belo Horizonte, MG, Brazil

**Márcio Marcarenhas,** Universidade Federal do Piauí, teresina, PI, Brazil

**Marco Aurelio Galletta,** Universidade de São Paulo, São Paulo, SP, Brazil

**Marco Aurélio Pinho de Oliveira,** Universidade do Estado do Rio de Janeiro, Rio de Janeiro, RJ, Brazil

**Marcos Dias,** Instituto Fernandes Figueira, Rio de Janeiro, RJ, Brasil.

**Marcos Masaru Okido,** Universidade de São Paulo, São Paulo, SP, Brazil

**Marcos Messina,** Universidade São Paulo, São Paulo, SP, Brazil

**Marcos Nakamura Pereira,** Fundação Oswaldo Cruz, Manguinhos, RJ, Brazil

**Marcos Pereira Santos,** Universidade Federal da Bahia, Salvador, BA, Brazil

**Marcos Sousa,** Universidade Estadual de Campinas, Campinas, SP, Brazil

**Marcos Tcherniakovsky,** Faculdade de Medicina do ABC, Santo André, SP, Brazil

**Marcos Wengrover Rosa,** Hospital Moinhos de Vento, Porto Alegre, RS, Brazil

**Marcus Silva,** Universidade Federal do Amazonas, Manaus, AM, Brazil

**Maria Aparecida Nagai,** Universidade de São Paulo, São Paulo, SP, Brazil

**Maria Augusta Bortolini,** Universidade Federal de São Paulo, São Paulo, SP, Brazil

**Maria Brizot,** Universidade de São Paulo, São Paulo, SP, Brazil

**Maria Camargo,** Mayo Clinic in Arizona, Arizona, USA

**Maria Candida Baracat,** Universidade de São Paulo, São Paulo, SP, Brazil

**Maria Carolina Szymansky,** Universidade Estadual de Campinas, Campinas, SP, Brazil

**Maria Celeste Wender,** Universidade Federal do Rio Grande do Sul, Porto Alegre, RS, Brazil

**Maria Célia Mendes,** Universidade de São Paulo, Ribeirão Preto, SP, Brazil

**Maria de Lourdes Brizot,** Universidade de São Paulo, São Paulo, SP, Brazil

**Maria de Lourdes da Silva,** Universidade Federal do Rio Grande do Norte, Natal, RN, Brazil

**Maria Elisabeth Moreira,** Fundacao Oswaldo Cruz, Rio de Janeiro, RJ, Brazil

**Maria Elisabetta Coccia,** Università di Firenze, Firenze, Italy

**Maria Helena Bastos,** Universidade São Paulo, São Paulo, SP, Brazil

**Maria Jose Nogueira,** Universidade de Évora, Évora, Portugal

**Maria José Soares Júnior,** Universidade de São Paulo, São Paulo, SP, Brazil

**Maria Julia Calas,** Diagnósticos da América SA, Clínica de Diagnóstico por Imagem, Rio de Janeiro, RJ, Brazil

**Maria Julia Miele,** Universidade Estadual de Campinas, Campinas, SP, Brazil

**Maria Laura Costa,** Universidade Estadual de Campinas, Campinas, SP, Brazil

**Maria Laura Costa do Nascimento,** Universidade Estadual de Campinas, Campinas, SP, Brazil

**Maria Lucia Fleiuss de Farias,** Universidade Federal do Rio de Janeiro, Rio de Janeiro, RJ, Brazil

**Maria Lucia Lima,** Universidade de São Paulo, Ribeirão Preto, SP, Brazil

**Maria Lucia Oliveira Ferrari,** (Consultório próprio), São Paulo, SP, Brazil

**Maria Lucia Oppermann,** Universidade Federal do Rio Grande do Sul, Porto Alegre, RS, Brazil

**Maria Lúcia Rocha,** Universidade Federal do Rio Grande do Sul, Porto Alegre, RS, Brazil

**Maria Mendes,** Universidade de São Paulo, Ribeirão Preto, SP, Brazil

**Maria Pilar Diz,** Universidade de São Paulo, Ribeirão Preto, SP, Brazil

**Maria Pilar Estevez Diz,** Universidade de São Paulo, São Paulo, SP, Brazil

**Maria Respondek-Liberska,** Institute of Polish Mother’s Health Center, Łódź, Poland

**Maria Rita Bortolotto,** Universidade de São Paulo, São Paulo, SP, Brazil

**Maria Rita Lerri,** Universidade de São Paulo, Ribeirão Preto, SP, Brazil

**Maria Teresa Sanseverino,** Hospital das Clínicas, Porto Alegre, RS, Brazil

**Mariana Alice,** Universidade Estadual Paulista Julio de Mesquita Filho, Botucatu, SP, Brazil

**Mariana Andres,** Hospital Beneficencia Portuguesa de São Paulo, São Paulo, SP, Brazil

**Mariana Arruda Silva,** Universidade de São Paulo, São Paulo, SP, Brazil

**Mariana Azevedo Carvalho,** Universidade de São Paulo, São Paulo, SP, Brazil

**Mariana Seabra,** Universidade Federal de Minas Gerais, Belo Horizonte, MG, Brazil

**Mariane Barbieri,** Universidade Estadual de Campinas, Campinas, SP, Brazil

**Mariane Nadai,** Universidade de São Paulo, São Paulo, SP, Brazil

**Mariane Nunes de Nadai,** Hospital das Clínicas, Faculdade de Medicina, Universidade de São Paulo, Ribeirão Preto, SP, Brazil

**Marianna Brock,** Universidade do Estado de Amazonas, Manaus, AM, Brazil

**Mariano Gomes,** Hospital Israelita Albert Einstein, São Paulo, SP, Brazil

**Mariano Tamura Vieira Gomes,** Hospital Israelita Albert Einstein, São Paulo, SP, Brazil

**Marilene Vale,** Universiade Federal de Minas Gerais, Belo Horizonte, MG, Brazil

**Marilza Rudge,** Universidade Estadual Paulista Julio de Mesquita Filho, Botucatu, SP, Brazil

**Marina Zamith,** CETRUS, São Paulo, SP, Brazil

**Mario Dias Correa Junior,** Universidade Federal de Minas Gerais, Belo Horizonte, MG, Brazil

**Mario Henrique Burlacchini de Carvalho,** Universidade de São Paulo, São Paulo, SP, Brazil

**Mario Julio Franco,** Universidade Federal de Santa Catarina, Florianópolis, SC, Brazil

**Marisa Pinhata,** Universidade de São Paulo, Ribeirão Preto, SP, Brazil

**Marli Duarte,** Universidade Estadual Paulista Julio de Mesquita Filho, Botucatu, SP, Brazil

**Marta Finotti,** Universidade Federal de Goias, Goiânia, GO, Brazil

**Marvyn Sacramento,** Faculdade Social da Bahia, Salvador, BA, Brazil

**Mary Nakamura,** Universidade Federal de São Paulo, São Paulo, SP, Brazil

**Matias Vieira,** King’s College London, London, United Kingdom

**Maurício Abrão,** Universidade de São Paulo, São Paulo, SP, Brazil

**Mauro Sancovski,** Faculdade de Medicina do ABC, Santo André, SP, Brazil

**Max Mano,** Grupooncoclinicas, Porto Alegre, RS, Brazil

**Maximiliano Guerra,** Universidade Federal de Juiz de Fora, Juiz de Fora, MG, Brazil

**Melânia Maria Ramos de Amorim,** Universidade Federal de Campina Grande, Campina Grande, PB, Brazil

**Melissa Premaor,** Universidade Federal de Minas Gerais, Belo Horizonte, MG, Brazil

**Michael Auerbach,** HemOnc

**Michele Da Broi,** Universidade de São Paulo, Ribeirão Preto, SP, Brazil

**Michele Gomes Da Broi,** Universidade de São Paulo, Ribeirão Preto, SP, Brazil

**Michele Moreira,** Universidade Federal do Rio Grande do Sul, Porto Alegre, RS, Brazil

**Michele Pedrosa,** Universidade Federal do Rio de Janeiro, Rio de Janeiro, RJ, Brazil

**Michelle Discacciati,** Universidade Estadual de Campinas, Campinas, SP, Brazil

**Miguel Sanchéz Polán,** Universidad Politecnica de Madrid, Madrid, Espanha

**Mila Salcedo,** Universidade Federal de Ciências da Saúde de Porto Alegre, Porto Alegre, RS, Brazil

**Milena Bastos Brito,** Escola Bahiana de Medicina e Saúde Pública, Salvador, BA, Brazil

**Milene Carrilho,** Universidade Federal de São Paulo, São Paulo, SP, Brazil

**Millena Jammal,** Universidade Federal do Triângulo Mineiro, Uberaba, MG, Brazil

**Mirela Jimenez,** Hospital Femina, Porto Alegre, RS, Brazil

**Mitra Eftekhariyazdi,** Sabzevar University of Medical Sciences, Sabzevar, Asad Abadi, Iran

**Mohammad Mohammadianpanah,** Shiraz University of Medical Sciences, Iran

**Monica Malta,** University of Toronto, Toronto, Canadá

**Monica Reis,** Hospital Municipal de Alto Araguaia, Alto Araguaia, MT, Brazil

**Montejo Rocio,** Universidade de Gotemburgo, Gothenburg, Suécia

**Murilo Borges,** São Camilo Saúde, São Paulo, SP, Brazil

**Murilo Soares,** Universidade de São Paulo, Ribeirão Preto, SP, Brazil

**Nadia Stela Reis,** Universidade Federal do Mato Grosso do Sul, Campo Grande, MS, Brazil

**Narayana Santana,** Faculdade de Medicina de Jundiai, Jundiaí, SP, Brazil

**Natalia Zavattiero,** Instituto Verhum, Brasília, DF, Brazil

**Natan Feter,** Universidade Federal de Pelotas, Pelotas, RS, Brazil

**Nathalie Bravo-Valenzuela,** Universidade Federal de São Paulo, São Paulo, SP, Brazil

**Neide Aparecida Boldrini Tosato,** Universidade Federal do Espirito Santo, Vitória, ES, Brazil

**Neide Boldrini,** Universidade Federal do Espírito Santo, Vitória, ES, Brazil

**Neila Speck,** Universidade Federal de São Paulo, São Paulo, SP, Brazil

**Nelson Sass,** Universidade Federal de São Paulo, São Paulo, SP, Brazil

**Newton de Carvalho,** Universidade Federal do Paraná, Curitiba, PR, Brazil

**Newton Eduardo Busso,** Faculdade de Ciências Médicas da Santa Casa de São Paulo, São Paulo, SP, Brazil

**Newton Tomio Miyashita,** Amparo Maternal, São Paulo, SP, Brazil

**Nicola Fratelli,** University of Brescia, Brescia, Italy

**Nicoli Serquiz,** Universidade Federal do Rio Grande do Norte, Natal, RN, Brazil

**Nilma Antas Neves,** Universidade Federal da Bahia, Salvador, BA, Brazil

**Nilson Szylit,** Hospital Israelita Albert Einstein, São Paulo, SP, Brazil

**Odette Sanchez,** Universidade Estadual de Campinas, Campinas, SP, Brazil

**Oksana Shynlova,** Sinai Health System, LTRI, Toronto, Canadá

**Olímpio Barbosa Morais Filho,** Universidade de Pernambuco, Recife, PE, Brazil

**Olivia Cardenas-Trowers,** University of Louisville, Louisville, USA

**Omero Benedicto Poli Neto,** Universidade de São Paulo, Ribeirão Preto, SP, Brazil

**Osmar Rangel Neto,** Universidade Federal de São Paulo, São Paulo, SP, Brazil

**Otávio Alabarse,** Universidade Estadual de Campinas, Campinas, SP, Brazil

**Paolo Cavoretto,** Università Vita-Salute San Raffaele, Milano, Italy

**Patrícia El Beitune,** Universidade Federal de Ciências da Saúde de Porto Alegre, RS, Brazil

**Patrícia Lopes,** Universidade Federal Fluminense, Niteroi, RJ, Brazil

**Patricia Melli,** Universidade de São Paulo, Ribeirão Preto, SP, Brazil

**Patrícia Moreira Gomes,** Universidade de São Paulo, Ribeirão Preto, SP, Brazil

**Patrícia Rehder,** Universidade Estadual de Campinas, Campinas, SP, Brazil

**Patrícia Teixeira,** Universidade Federal de Minas Gerais, Belo Horizonte, MG, Brazil

**Paula Andrea de Albuquerque Salles Navarro,** Universidade de São Paulo, Ribeirão Preto, SP, Brazil

**Paula Clara Santos,** Instituto Politécnico do Porto, Porto, Portugal

**Paula Elias,** Universidade de São Paulo, Ribeirão Preto, SP, Brazil

**Paula Machado,** Universidade Federal do Rio Grande do Norte, Natal, RN, Brazil

**Paula Terraciano,** Hospital de Clinicas de Porto Alegre, Porto Alegre, RS, Brazil

**Paulo Afonso Beltrame,** Universidade Federal de Santa Maria, Santa Maria, RS, Brazil

**Paulo Henrique Manso,** Universidade de São Paulo, Ribeirão Preto, SP, Brazil

**Paulo Kawano,** Universidade Estadual Paulista Julio de Mesquita Filho, Botucatu, SP, Brazil

**Paulo Meade,** Universidad Autónoma de San Luis Potosí, Mexico

**Paulo Nassar de Carvalho,** Instituto Fernandes Figueira, Rio de Janeiro, RJ, Brasil.

**Paulo Roberto Dutra Leão,** Universidade de Cuiabá, Cuiabá, MT, Brazil

**Pedro Castro,** Faculdades Souza Marques, Rio de Janeiro, RJ, Brazil

**Pedro do Valle Teichmann,** Universidade Federal do Rio Grande do Sul, Porto Alegre, RS, Brazil

**Pedro Monteleone,** Centro de Reprodução Humana Monteleone, São Paulo, SP, Brazil

**Pedro Pires,** Universidade de Pernambuco, Recife, PE, Brazil

**Pedro Soler Coltro,** Universidade de São Paulo, São Paulo, SP, Brazil

**Penelope Marinho,** Universidade Federal do Rio de Janeiro, Rio de Janeiro, RJ, Brazil

**Penny Sheehan,** The Royal Women’s Hospital, Parkville Victoria, Australia

**Petr Stourac,** Masaryk University, Brun, Chéquia

**Pietro Grussu,** National Health System, Consultorio Familiare, South Padua District, Azienda ULSS 6 Euganea, Veneto Region

**Pisake Lumbiganon,** Khon Kaen University, Obstetrics and Gynecology, Khon Kaen, Tailândia

**Priscila Rezeck Nunes,** Universidade Estadual Paulista Júlio de Mesquita Filho, Botucatu, SP, Brazil

**Priscila Silva,** Universidade Estadual de Campinas, Campinas, SP, Brazil

**Qin Xiang Ng,** MOH Holdings Pte Ltd, 1 Maritime Square, Singapura

**Rabih Chaoui,** Feindiagnostik, Ankara,Turkey

**Rafael Bruns,** Universidade Federal do Parana, Curitiba, PR, Brazil

**Rafael Cortés-Charry,** Hospital Universitario de Caracas, Caracas, Venezuela

**Rafael Galvão,** Universidade Estadual de Campinas, Campinas, SP, Brazil

**Rafael Moroni,** Universidade Estadual do Oeste do Parana, Cascavel, PR, Brazil

**Rafael Rosa,** Universidade Federal de Ciências da Saúde de Porto Alegre, Porto Alegre, RS, Brazil

**Rafaela Alkmin Costa,** Universidade de São Paulo, São Paulo, SP, Brazil

**Rafaela Costa,** Universidade de São Paulo, São Paulo, SP, Brazil

**Rafaella Petracco,** Pontifícia Universidade Católica do Rio Grande do Sul, Porto Alegre, RS, Brazil

**Raquel Arruda,** Universidade Federal de São Paulo, São Paulo, SP, Brazil

**Raquel Coelho,** Universidade Federal do Ceara, Fortaleza, CE, Brazil

**Raquel Rivero,** Universidade Federal do Rio Grande do Sul, Porto Alegre, RS, Brazil

**Rebecca Sotelo,** Hospital Federal de Ipanema, Rio de Janeiro, RJ, Braszil

**Regina Amélia Aguiar,** Universidade Federal de Minas Gerais, Belo Horizonte, MG, Brazil

**Reginaldo Freitas-Júnior,** Instituto de Ensino e Pesquisa Alberto Santos Dumont, São Paulo, SP, Brazil

**Reginaldo Lopes,** Instituto de Assistência Médica ao Servidor Público Estadual de São Paulo, São Paulo, SP, Brazil

**Regis Kreitchmann,** Universidade Federal de Ciência da Saúde de Porto Alegre, Porto Alegre, RS, Brazil

**Reinaldo Figueiroa,** Saint Francis Hospital, Monroe, LA, USA

**Renata Cruz Soares de Azevedo,** Universidade Estadual de Campinas, Campinas, SP, Brazil

**Renata Freitas,** Universidade Federal de Juiz de Fora, Juiz de Fora, MG, Brazil

**Renata Ribeiro,** Tribunal de Justiça do Distrito Federal e Territorios, Brasília, DF, Brazil

**Renata Souza,** Universidade Estadual de Campinas, Campinas, SP, Brazil

**Renato Fraietta,** Universidade Federal de São Paulo, São Paulo, SP, Brazil

**Renato Hosoume,** Universidade de São Paulo, Ribeirão Preto, SP, Brazil

**Renato Moretti-Marques,** Hospital Israelita Albert Einstein, São Paulo, SP, Brazil

**Renato Passini Júnior,** Universidade Estadual de Campinas, Campinas, SP, Brazil

**Renato Sa,** Universidade Federal Fluminense, Niterio, RJ, Brazil

**Renato Sousa,** Universidade Estadual de Campinas, Campinas, SP, Brazil

**Renato Torresan,** Universidade Estadual de Campinas, Campinas, SP, Brazil

**Rene Vieira,** Hospital de Cancer de Barretos, Barretos, SP, Brazil

**Reyna Samano,** UAM Xochimilco, UIA, EDN.: Ciudad de México, México

**Rha Maroyi,** Université Evangélique en Afrique, República Democrática do Congo

**Ricardo Barini,** Universidade Estadual de Campinas, Campinas, SP, Brazil

**Ricardo Carvalho Cavalli,** Universidade de São Paulo, Ribeirão Preto, SP, Brazil

**Ricardo Cobucci,** Universidade Potiguar, Teresina, PI, Brazil

**Ricardo dos Reis,** Hospital de Amor, Barretos, SP, Brazil

**Ricardo Lasmar,** Universidade Federal Fluminense, Niteroi, RJ, Brazil

**Ricardo Marinho,** Faculdade Ciências Médicas de Minas Gerais, Belo Horizonte, MG, Brazil

**Ricardo Reis,** Hospital de Cancer de Barretos, Barretos, SP, Brazil

**Ricardo Savaris,** Universidade Federal do Rio Grande do Sul, Porto Alegre, RS, Brazil

**Ricardo Tedesco,** Faculdade de Medicina de Jundiai, Jundiaí, SP, Brazil

**Riccardo Lacchini,** Universidade de São Paulo, Ribeirão Preto, SP, Brazil

**Rigmor Berg,** University of Georgia, Athens, USA

**Rita de Cássia Maio Dardis,** Universidade Federal de São Paulo, São Paulo, SP, Brazil

**Rita Zanine,** Universidade Federal do Parana, Curitiba, PR, Brazil

**Ritu Mogra,** Monash University, Melbourne, Victoria, Australia

**Rivia Lamaita,** Universidade Federal de Minas Gerais, Belo Horizonte, MG, Brazil

**Riza Madazli,** Universidade de Istambul, Istambul, Turkey

**Roberta Bgeginski,** Universidade de Western Ontario, London, Canadá

**Robinson Dias de Medeiros,** Universidade Federal do Rio Grande do Norte, Natal, RN, Brazil

**Rodolfo Pacagnella,** Universidade Estadual de Campinas, Campinas, SP, Brazil

**Rodolfo Strufaldi,** Faculdade de Medicina do ABC, Santo André, SP, Brazil

**Rodrigo Alves Ferreira,** Universidade Federal de São Carlos, São Carlos, SP, Brazil

**Rodrigo Bernardes Cardoso,** Universidade Federal de Ciências da Saúde de Porto Alegre, Porto Alegre, RS, Brazil

**Rodrigo Gonçalves,** Universidade de São Paulo, São Paulo, SP, Brazil

**Rodrigo Lisboa,** Universidade de São Paulo, São Paulo, SP, Brazil

**Rodrigo Rocha,** Instituto Da Vinci, Toledo, PR, Brazil

**Rodrigo Ruano,** Mayo Clinic, Department of Obstetrics and Gynecology, Phoenix, Arizona, USA

**Rodrigo Santana,** Universidade de São Paulo, São Paulo, SP, Brazil

**Roger Bouillon,** KU Leuven, Leuven, Belgium

**Rogério Leão,** Universidade Estadual de Campinas, Campinas, SP, Brazil

**Romulo Negrini,** Faculdade de Ciências Médicas da Santa Casa de São Paulo, São Paulo, SP, Brazil

**Roney Signorini,** Universidade Federal de São Paulo, São Paulo, SP, Brazil

**Rosa Maria Domingues,** Instituto Nacional de Infectologia Evandro Chagas, Rio de Janeiro, RJ, Brazil

**Rosana dos Reis,** Universidade de São Paulo, Ribeirão Preto, SP, Brazil

**Rosana Maria dos Reis,** Universidade de São Paulo, Ribeirão Preto, SP, Brazil

**Rosane Figueiredo Alves,** Universidade Federal de Goias, Goiania, GO, Brazil

**Rosekeila Nomelini,** Universidade Federal do Triângulo Mineiro, Uberaba, MG, Brazil

**Roseli Nomura,** Universidade Federal de São Paulo, São Paulo, SP, Brazil

**Rosemeire Albuquerque,** Universidade de São Paulo, São Paulo, SP, Brazil

**Rosemeire Sartori de Albuquerque,** Universidade de São Paulo, São Paulo, SP, Brazil

**Rosiane Mattar,** Universidade Federal de São Paulo, São Paulo, SP, Brazil

**Roxana Knobel,** Universidade Federal de Santa Catarina, Florianópolis, SC, Brazil

**Rozi Aryananda,** Airlangga University, Indonésia

**Rubens Tavares,** Universidade Federal de Minas Gerais, Belo Horizonte, MG, Brazil

**Rubmeide Gallo,** Universidade Federal do Parana, Curitiba, PR, Brazil

**Rui Ferriani,** Universidade de São Paulo, Ribeirão Preto, SP, Brazil

**Rumyana Markovska,** University of Sofia, Sofia, Bulgária

**Sabas Vieira,** Oncocentro, São Paulo, SP, Brazil

**Salomone Di Saverio,** AUSL di Bologna

**Salvatore Caruso,** Universidade da Catânia, Catania, Italy

**Samantha Conde,** Hospital Geral da Santa Casa da Misericordia do Rio de Janeiro, Rio de Janeiro, RJ, Brazil

**Samira El Maerrawi Tebecherane Haddad,** Universidade Estadual de Campinas, Campinas, SP, Brazil

**Samira Haddad,** Universidade Estadual de Campinas, Campinas, SP, Brazil

**Sandra Fonseca,** Universidade Federal Fluminense, Niterio, RJ, Brazil

**Sandra Regina Campos Teixeira,** Hospital Vera Cruz, Radiologia e Diagnóstico por Imagem, Campinas, SP, Brazil

**Sandra Scalco,** Universidade Federal do Rio Grande do Sul, Porto Alegre, RS, Brazil

**Sandra Teixeira,** Clínica CDE - Diagnóstico por Imagem, Campinas, SP, Brazil

**Sanne Anjel,** Aarhus University, Denmark & Molde University College, Norway

**Sara Solha,** Pontifícia Universidade Católica de São Paulo, São Paulo, SP, Brazil

**Sarah Rominki,** University of Michigan, Michigan, USA

**Sávia Francisco Lopes Dias,** Universidade Federal de São Paulo, São Paulo, SP, Brazil

**Sckarlet Biancolin,** Universidade de São Paulo, São Paulo, SP, Brazil

**Sebastião de Medeiros,** Universidade Federal do Mato Grosso, Cuiabá, MT, Brazil

**Sebastião Freitas de Medeiros,** Universidade Federal de Mato Grosso, Cuiabá, MT, Brazil

**Selmo Geber,** Universidade Federal de Minas Gerais, Belo Horizonte, MG, Brazil

**Sergio Brasileiro,** Universidade Federal de São Paulo, São Paulo, SP, Brazil

**Sergio Hecker Luz,** Pontifícia Universidade Católica do Rio Grande do Sul, Porto Alegre, RS, Brazil

**Sergio Kobayashi,** Universidade Federal de São Paulo, São Paulo, SP, Brazil

**Sergio Makabe,** Universidade Municipal de São Caetano do Sul, São Caetano do Sul, SP, Brazil

**Sergio Mancin Nicolau,** Universidade Federal de São Paulo, São Paulo, SP, Brazil

**Sergio Marba,** Universidade Estadual de Campinas, Campinas, SP, Brazil

**Sérgio Martins Costa,** Universidade Federal do Rio Grande do Sul, Porto Alegre, RS, Brazil

**Sergio Okano,** Universidade de São Paulo, São Paulo, SP, Brazil

**Sergio Podgaec,** Universidade de São Paulo, São Paulo, SP, Brazil

**Seval Ozgu-Erdinc,** University of Health Sciences, Ankara, Turkey

**Sherif Ashoush,** Ain Shams University, Cairo, Egypt

**Sheyla Rocha,** Universidade Estadual de Campinas, Campinas, SP, Brazil

**Sheyla Silveira,** Hospital Universitário Polydoro Ernani de São Thiago, Florianópolis, SC, Brazil

**Shoubhi Kahhale,** Universidade de São Paulo, São Paulo, SP, Brazil

**Silvana Maria Quintana,** Universidade de São Paulo, Ribeirão Preto, SP, Brazil

**Silvane Portela,** Universidade Federal da Fronteira Sul, Passo Fundo, RS, Brazil

**Silvia D’Ippolito,** Università Cattolica del Sacro Cuore, Rome, Italy

**Sílvia Piza,** Irmandade da Santa Casa de Misericordia de Sao Paulo, São Paulo, SP, Brazil

**Silvia Rabelo,** Universidade Federal de Goias, Goiânia, GO, Brazil

**Silvia Setubal,** Universidade Estadual de Campinas, Campinas, SP, Brazil

**Silvio Martinelli,** Universidade de São Paulo, São Paulo, SP, Brazil

**Simone Miranda,** Universidade Estadual do Piauí, Floriano, PI, Brazil

**Simony Nascimento,** Universidade Federal do Ceara, Fortaleza, CE, Brazil

**Sirlei Morais,** Universidade Estadual de Campinas, Campinas, SP, Brazil

**Solange Frid,** Pontifícia Universidade Católica do Rio de Janeiro, RJ, Brazil

**Sophie Françoise Mauricette Derchain,** Universidade Estadual de Campinas, Campinas, SP, Brazil

**Sóstenes Postigo,** Faculdade de Ciencias Medicas da Santa Casa de Sao Paulo, São Paulo, São Paulo, SP, Brazil

**Stany Rodrigues de Paula,** Universidade de São Paulo, São Paulo, SP, Brazil

**Stefan Kane,** The Royal Women’s Hospital, Maternal Fetal Medicine, Australia

**Stefania Triunfo,** Univerita Cattolica del Sacro Cuore, Milano, Italy

**Stephanie L. Martin,** University of North Carolina, Chapel Hill, North Carolina, USA

**Sue Sun,** Universidade Federal de São Paulo, São Paulo, SP, Brazil

**Sultana Tahmina,** Melmaruvathur Adhiparasakthi Institute of Medical Sciences and Research, Tamil Nadu, Índia

**Sun Lee,** Boston University, Boston, USA

**Susana Ramalho,** Universidade Estadual de Campinas, Campinas, SP, Brazil

**Suzana Pessini,** Universidade Federal do Rio Grande do Sul, Porto Alegre, RS, Brazil

**Suzana Pires do Rio,** Fundação Hospitalar do Estado de Minas Gerais, Belo Horizonte, MG, Brazil

**Suzana Turra,** Complexo Hospitalar Santa Casa de Misericórdia de Porto Alegre, Porto Alegre, RS, Brazil

**Suzane Serruya,** Organização Pan-Americana da Saúde, Washignton, DC, USA

**Sylvia Hayashida,** Universidade de São Paulo, São Paulo, SP, Brazil

**Tadahiko Shien,** Okayama University Hospital, Okayama, Japão

**Tadeu Coutinho,** Universidade Federal de Juiz de Fora, Juiz de Fora, MG, Brazil

**Tainá Pezzin,** Universidade de São Paulo, São Paulo, SP, Brazil

**Taina Sanches,** Universidade de São Paulo, Riberião Preto, SP, Brazil

**Talira Colombo,** Universidade Federal de Ciências da Saúde de Porto Alegre, RS, Brazil

**Tarciana Almeida,** Instituto de Medicina Integral Professor Fernando Figueira, Recife, PE, Brazil

**Tea Mladenic,** University of Rijeka Faculty of Medicine, Rijeka, Croácia

**Tezer Kutluk,** Hacettepe University, Ancar, Turkey

**Thais Gozzo,** Universidade de São Paulo, Ribeirão Preto, SP, Brazil

**Thais Lourenço,** Universidade de São Paulo, São Paulo, SP, Brazil

**Thais Rangel Bousquet Carrilho,** Universidade Federal do Rio de Janeiro, Rio de Janeiro, RJ, Brazil

**Thais Silva,** Universidade Estadual de Campinas, Campinas, SP, Brazil

**Thales Paulo Batista,** Universidade Federal de Pernambuco, Recife, PE, Brazil

**Thalita Berteli,** Kofinas Fertility Group, Ribeirão Preto, SP, Brazil

**Théo Lerner,** Universidade Estadual do Piaui, Floriano, PI, Brazil

**Thiago Apolinário,** Universidade de São Paulo, Ribeirão Preto, SP, Brazil

**Thorsten Braun,** Universitätsmedizin Berlin, Berlin, Alemanha

**Ticiana Mara,** Universidade Estadual de Campinas, Campinas, SP, Brazil

**Tim Lee,** Monash University, Melbourne, Victoria, Australia

**Titus Beyuo,** Korle Bu Teaching Hospital, Korlebu, Gana

**Tony Bazi,** American University of Beirut, Beirut, Lebanon

**Toshiyuki Hata,** Kagawa University, Takamatsu, Kagawa, Japão

**Unyime Ituk,** University of Iowa, Iowa City, Estados Unidos

**Valdair Muglia,** Universidade de São Paulo, Ribeirão Preto, SP, Brazil

**Valdes Roberto Bollela,** Universidade de São Paulo, Ribeirão Preto, SP, Brazil

**Valentino Magno,** Universidade Federal do Rio Grande do Sul, Porto Alegre, RS, Brazil

**Valéria Gomes,** Universidade de São Paulo, São Paulo, SP, Brazil

**Valéria Pontes,** Universidade Federal do Pará, Belém, PA, Brazil

**Vania Meira e Siqueira Campos,** Universidade Federal de Goias, Goiânia, GO, Brazil

**Vardeli Alves de Moraes,** Universidade Federal de Goiás, Goiânia, GO, Brazil

**Varmita Abdo,** Universidade de São Paulo, São Paulo, SP, Brazil

**Vasilios Pergialiotis,** National and Kapodistrian University of Athens, Atenas, Grécia

**Vera Borges,** Universidade Estadual Paulista Julio de Mesquita Filho, Botucatu, SP, Brazil

**Vicente Antonello,** Hospital Femina, São Paulo, SP, Brazil

**Victor Campos,** Universidade de São Paulo, Ribeirão Preto, SP, Brazil

**Victor Hugo Saucedo Sanchez,** Universidade Federal de São Paulo, São Paulo, SP, Brazil

**Vinicius Aniceto,** Universidade de São Paulo, Ribeirão Preto, SP, Brazil

**Vinicius Mochini,** Universidade Estadual de Campinas, Campinas, SP, Brazil

**Vinicius Oliviero,** Faculdade de Tecnologia de São Paulo, São Paulo, SP, Brazil

**Virgilio Sacchini,** Memorail Sloan Kettering Cancer Center, New York, USA

**Viviane Cardoso,** Universidade de São Paulo, Ribeirão Preto, SP, Brazil

**Volney Câmara,** Universidade Federal do Rio de Janeiro, Rio de Janeiro, RJ, Brazil

**Wagner Gonçalves,** Universidade Federal de São Paulo, São Paulo, SP, Brazil

**Waldir Modotti,** Instituto de Atendimento Médico Hospitalar, Ginecologia e Endoscopia, Assis, SP, Brazil

**Walquiria Primo,** Universidade de Brasília, Brasília, DF, Brazil

**Wanuzia Miranda,** Faculdades de Enfermagem e Medicina Nova Esperanca, João Pessoa, PB, Brazil

**Wellington Martins,** SEMEAR Fertilidade, Ribeirão Preto, SP, Brazil

**Wilson Ayach,** Universidade Federal de Mato Grosso do Sul, Campo Grande, MS, Brazil

**Yanyun Wang,** Sichuan University, Si Chuan Sheng, China

**Yara Furtado,** Universidade Federal do Rio de Janeiro, Rio de Janeiro, RJ, Brazil

**Zilma Reis,** Universidade Federal de Minas Gerais, Belo Horizonte, MG, Brazil

**Zsuszanna Jarmy-di-Bella,** Universidade Federal de São Paulo, São Paulo, SP, Brasil

